# Reliability and validity of the mandarin version of the supportive care needs survey short-form (SCNS-SF34) and the head and neck cancer-specific supportive care needs (SCNS-HNC) module

**DOI:** 10.1186/s12913-020-05793-3

**Published:** 2020-10-16

**Authors:** Jianxia Lyu, Li Yin, Ping Cheng, Bin Li, Shanshan Peng, Chunlian Yang, Jing Yang, Haixin Liang, Qinghua Jiang

**Affiliations:** 1 Head & Neck Department of Radiation Oncology of Sichuan Cancer Hospital & Institute, Sichuan Cancer Center, School of Medicine, University of Electronic Science and Technology of China, Radiation Oncology Key Laboratory Of Sichuan Province, Chengdu, China; 2 Nursing department of Sichuan Cancer Hospital & Institute, Sichuan Cancer Center, School of Medicine, University of Electronic Science and Technology of China, Radiation Oncology Key Laboratory Of Sichuan Province, 4th Section of Renmin South Road, Chengdu, 610041 Sichuan Province People’s Republic of China

**Keywords:** Head and neck cancer, Support care needs, Reliability, Validity, SCNS-HNC scale

## Abstract

**Background:**

This study aimed to translate the English version of the supportive care needs scale of head and neck cancer patients (SCNS-HNC) questionnaire into Mandarin and to test the reliability and validity of the SCNS-SF34 and SCNS-HNC module in head and neck cancer patients.

**Methods:**

The Mandarin version of the Supportive Care Needs Survey Short-Form (SCNS-SF34) and SCNS-HNC scales were used to assess 206 patients with head and neck cancer in Chengdu, China. Among them, 51 patients were re-tested 2 or 3 days after the first survey. The internal consistency of the scale was evaluated by Cronbach’s alpha coefficient, the retest reliability of the scale was evaluated by retest correlation coefficient r, the structural validity of the scale was evaluated by exploratory factor analysis, and the ceiling and floor effects of the scale were evaluated.

**Results:**

The Mandarin version of the SCNS-HNC had Cronbach’s alpha coefficients greater than 0.700 (0.737 ≤ 0.962) for all of the domains. Except for the psychological demand dimension (r = 0.674) of the SCNS-SF34 scale, the retest reliability of the other domains was greater than 0.8. Three common factors were extracted by exploratory factor analysis, and the cumulative variance contribution rate was 64.39%.

**Conclusions:**

The Mandarin version of the SCNS-SF34 and SCNS-HNC demonstrated satisfactory reliability and validity and is able to measure the supportive care needs of Chinese patients with head and neck cancer.

**Trial registration:**

ChiCTR, ChiCTR1900026635. Registered 16 October 2019- Retrospectively registered.

## Background

Cancer is one of the leading causes of death worldwide [1]; in recent years, the morbidity and mortality of cancer in China have also increased [2]. Research shows [3] that in addition to medicine, surgery, chemoradiotherapy and other treatment measures, cancer patients and their families have a need for supportive care in the management of disease symptoms and side effects, especially information about medical decisions during illness and treatment. Patients with different types of cancer have different needs for supportive care [4]. According to incomplete statistics, there are more than 900,000 new cases of head and neck cancer (HNC) in the world every year [5], and HNC is the sixth most common cancer in the world today. China has a high incidence of head and neck cancer, with an annual incidence of approximately 15.34/100,000 [6], accounting for approximately 10% of all malignant cancers. Head and neck cancer anatomically include cancers of the head, face, ear, nose, throat, mouth, thyroid and other parts (from the base of the skull to supraclavicular region, excluding cervical vertebra) [7]. The head contains the eyes, ears, nose, tongue, throat and other important organs. Although the survival rate of patients with head and neck cancer has increased gradually with the improvement of medical technology, during treatment, patients often face a series of disease-related symptoms, such as nasal congestion, hoarse voice, difficulty chewing and other diseases, as well as a variety of treatment-related symptoms and functional disorders, such as taste loss, oral pain, radioactive mucositis/dermatitis, difficulty opening the mouth, and difficulty swallowing [8]. The head and face also have a great impact on the appearance and image of patients [9]. Additionally, patients are often faced with a large number of psychosocial problems, such as depression [10], stigma [11], economic difficulties [12], communication difficulties [13], death threats [14], and fear of recurrence [15], which seriously affect the quality of life of patients with head and neck cancer. In addition, approximately 30–50% of HNC patients are associated with varying degrees of malnutrition [16]. The Supportive Care Needs Survey-Head & Neck Cancer (SCNS-HNC) is an 11-item survey assessing HNC-specific needs. The original SCNS-HNC was developed and tested in the Dutch language in the Netherlands [17] and comprised two domains: HNC-specific functioning and lifestyle. The SCNS-HNC is intended to be used as a supplementary module to the core SCNS which is available in a 59-item long form (SCNS-LF59) [18] or 34-item short-form (SCNS-SF34) [19]. At present, there is no special assessment scale for the supportive care needs of head and neck cancer patients in China. And the linguistic and cultural differences between countries may affect psychometric characteristics of patient reported outcome measures [20]. The purpose of this study was to Sinicize the SCNS-HNC module and evaluate its reliability and validity in Chinese patients with head and neck cancer to provide a tool for evaluating the supportive care needs of Chinese patients with head and neck cancer.

## Methods

### Instruments

The SCNS-HNC module and technical documents were obtained from the original authors, And the module was translated into Mandarin through standard translation, back translation, cultural debugging and evaluation procedures by the EORTC Qol group [21].

### Translation procedure

The translation and back translation processes were divided into the following steps: (1) The forward translation was completed independently by two translators who are native speakers of Chinese. The translation results were named translation 1 and translation 2. Translator 1 was a master of medicine who knows relevant medical terms and can adjust terms from the perspective of clinical practice to ensure medical equivalence with the original scale. Translator 2, a master of English major without a medical background, mainly translated from the perspective of language to consider the language habits of the general public. (2) The synthesis was performed by an M.D. who was not involved in the “forward translation” and who conducted a comparative analysis of the two translated versions (translation 1 and translation 2). If there were any differences, three people discussed and coordinated with each other to decide on the translation version, namely, translation 3. (3) Back translation was then performed. Two translators proficient in English and Chinese independently back translated translation 3, forming back translation version 1 and back translation version 2. Back translation translator 1 was a master’s student; back translation translator 2 was a doctoral student. Neither of them knew or was informed of the content or purpose of the scale, and neither of them had a medical background to avoid information deviation and at the same time discover the hidden translation differences of the items in translation 3. Finally, a doctor with a master’s degree in medicine compared the translated English version with the original scale, determined the differences and modified the Mandarin version appropriately; then, the doctor gave it to the translation team for translation, compared it with the original scale, and repeated it until the English version was as similar as possible to the original scale.

### Cultural adaptation

According to the characteristics of the Chinese culture, some items in the scale were adjusted to fit the Chinese cultural background and context, which was called cultural adaptation. Cultural adaptation had the following two aspects: (1) The first aspect was expert consultation. Two chief physicians, deputy chief physicians and deputy chief nurses of head and neck cancer, 1 medical doctor with overseas study experience, 1 professional English teacher, 1 master of psychology, 1 statistical expert and all translators were invited to form the expert committee. Then, according to the habits and context of Chinese culture, the first draft of the SCNS-HNC scale in Chinese was adjusted from four aspects: semantic equivalence, idiomatic equivalence, empirical equivalence and conceptual equivalence. For example, item 4 “inform nutrition status” was adjusted to “need to know nutrition knowledge”. The revised version 1 of the Chinese scale was formed after adjustment. (2) Then, preliminary investigation was performed. To ensure that the language of the scale was easy to understand and accept, 20 head and neck cancer patients whose native language is Chinese were selected from Sichuan Cancer Hospital for preliminary investigation. First, we explained the purpose and significance of the survey to the patient in detail, and in the patients completed the survey after providing informed consent. After the completion of the scale, each respondent participated in an interview for approximately 5–10 min, discussing whether the content of the scale contained any items with vague meaning that was difficult to understand. The understanding and feedback of the pre-respondents on the items were recorded, and version 1 of the Chinese scale was revised and proofread according to the answers to the interview questions. For example, item 9, “neck or shoulder movement disorder”, was modified to “support needs for shoulder and neck movement difficulty” to ensure easy understanding of the scale items, and the Mandarin version of the SCNS-HNC scale was finally formed.

### Sample and setting

The SCNS-HNC has a total of 11 items. According to the factor analysis requirements for sample size, the sample size should not be too small, and the proportion of sample size to basic variables should be above 5:1. The ideal sample size should be 10–25 times the number of variables [22], considering no response bias, and the sample size should be increased by 10%. Therefore, the ideal sample size = 11 * (10 ~ 25) * (1 + 10%) = 121 ~ 302.

A convenience sample of head and neck patients from Sichuan Cancer Hospital was recruited from inpatient treatment (surgery, chemotherapy and radiotherapy) and outpatient radiotherapy populations between January 2019 and May 2019. The inclusion criteria were as follows: (1) residents of mainland China, mainly living in mainland China from birth to participation in this study; (2) patients with a clinicopathologic diagnosis of head and neck carcinoma, including oral, pharynx, larynx, nasal cavity or large salivary adenocarcinoma (with a pathological or imaging diagnostic basis); (3) patients with an ability to understand and answer questions; (4) patients in whom the actual condition is known; (5) patients with an expected survival time ≥ 3 months; and (6) patients who provided informed consent to participate in this study voluntarily. The exclusion criteria were as follows: (1) patients with cognitive impairment or mental illness and (2) patients participating in other psychology-related clinical trials. All patients signed informed consent forms. This study was approved by the Sichuan Cancer Hospital Ethics Committee.

### Measures

#### General information questionnaire

The general information questionnaire part was self-designed based on a literature review and included two parts: (1) sociodemographic data: age, sex, nationality, education level, marital status, faith, role of caregiver, and long-term residence and (2) data on diseases and treatments, including cancer type, treatment and adverse reactions.

#### The mandarin version of 34-item short-form supportive care needs (SCNS-SF34)

The SCNS-SF34 is a scale to measure the needs of cancer patients and can be used to measure the supportive care needs of all cancer patients [19, 23]. The SCNS-SF34 contains 34 items in five dimensions: physical and daily living needs (5 items), psychological needs (10 items), sexual needs (3 items), health system and information needs (11 items), and patient care and support needs (5 items). The time frame for all entries is the past month, and the questions are answered using a Likert-type scale ranging from 1 to 5 points (1 = no need, not applicable; 2 = no need, satisfied; 3 = some need, low; 4 = some need, moderate; 5 = some need, high). The total score was calculated for each dimension. The original score was calculated according to the following conversion formula: Likert standard total score = (sum of the original scores for all entries in the dimension -m) × {100/(m × (k-1)}, where m is the number of items contained in the dimension, and K is the maximum score of each item, namely, 5. The Likert standard score was between 0 and 100 points: the higher the score, the higher the degree of need in this dimension [23, 24].

#### Mandarin version of the supportive care needs survey-head and neck Cancer (SCNS-HNC)

The Mandarin version of the SCNS-HNC module is the head and neck cancer module of the supportive care needs of cancer patients, which is used to determine the specific supportive care needs of patients with head and neck cancer. The SCNS-HNC should be combined with the Chinese SCNS-SF34 scale to evaluate the general needs of supportive care and the specific needs of head and neck cancer. The time frame and scoring method are the same as those of the SCNS-SF34.

### Data collection

The head nurse and a trained survey interviewer of each department (head and neck surgery ward, head and neck radiotherapy ward, outpatient radiotherapy department) identified eligible participants from the inpatient treatment (surgery, chemotherapy and radiotherapy) and outpatient radiotherapy list of Sichuan Cancer Hospital. Eligible patients were informed about the study by the head nurse or the interviewer and were assured that the quality of healthcare would not change regardless of whether they participated in the study. After giving informed consent, patients were asked to complete a self-report pen-and-paper questionnaire in the ward or waiting areas of the outpatient radiotherapy departments. If the patient had difficulty reading or writing (visual impairment, physical inconvenience), the interviewer would help to complete the survey by asking the question from the questionnaire. When the questionnaire was completed, the study investigators checked it thoroughly and clarified any missing responses with the patient immediately. The questionnaires were re-administered to the head and neck cancer patients 2–3 days later to evaluate the reliability of the scale.

### Statistical analysis

Excel (Microsoft Office Home and Student 2019) was used for data entry, and SPSS 21.0 software was used for data statistical analysis. Exploratory factor analysis (EFA, principal component analysis with oblique rotation) [25] was used to evaluate the structural validity of the scale. Bartlett’s spherical test and the KMO (Kaiser-Mayer-Olkin) test were required for the adaptability test before factor analysis. In factor analysis, it is required that the Chi^2^ value of Bartlett’s spherical test results be statistically significant (*P* < 0.05). The KMO test is used to investigate the partial correlation between variables, and it compares the simple correlation and partial correlation between variables. It is generally believed that KMO statistics > 0.5 can be used for factor analysis. Bartlett’s spherical test was mainly used to assess the correlations between variables, and *P* < 0.05 was used for factor analysis. The common factors that can represent the structure of the scale were extracted, and the cumulative variance contribution rate of the common factors was obtained. Each common factor was highly correlated with a group of specific variables. These common factors represent the basic structure of the scale, and the cumulative variance contribution rate reflects the cumulative effectiveness of the common factor to the scale [26]. The common factor can explain more than 40% of the variation. Moreover, each entry has a relatively high load value (≥ 0.4) on the corresponding factor, which is an ideal factor analysis result [27]. In the assessment of ceiling effects and floor effects, if more than 50% of the respondents reached the maximum or minimum extremum of each factor, it means that the factor has a ceiling effect and floor effect, respectively [28].

Cronbach’s alpha coefficient was used to evaluate the internal consistency of the scale, and test–retest reliability was measured by the Pearson correlation coefficient between the first and second assessments among the patients (r > 0.8 was considered acceptable). It is generally believed that Cronbach’s alpha coefficient > 0.7 indicates that the retest reliability of the scale is good [29].

Construct validity was assessed by correlations between the SCNS-SF34 and SCNS-HNC (scores < 0.4 indicating no undue overlap between the two questionnaires). The independent-samples *t* test (two-tailed) was used to compare the mean scores of each domain between two age groups. Based on previous studies, it was expected that younger patients (18–60 years) would report a higher level of need than older patients (> 60) in all domains except for the physiological domain [30]; males reported a higher level of sexual needs than females [31].

## Results

### Characteristics of participants

In this study, a total of 210 questionnaires were distributed, and 206 questionnaires were effectively recovered, with an effective recovery rate of 98.0%. Among the 206 patients, 125 were male and 81 were female. The mean age was 47.29 ± 12.678 years (age range 16 ~ 77 years). The general information and clinical information of the patients are shown in Table 1.

**Table 1:** Socio-demographic and clinical characteristics of the study samples(*n* = 206)

Characteristic	Number/Percent
Age in years mean (*SD*,range)	47.29 (12.678,16–77)
Gender	
Male	125 (60.68)
Female	81 (39.32)
Ethnics	
Han	195 (94.66)
Others	11 (5.34)
Educational status	
Primary school	34 (16.50)
Middle school	104 (50.49)
Polytechnic school	41 (19.90)
College and above	27 (13.11)
Marital status	
Single	28 (13.59)
Married/cohabiting	169 (82.04)
Divorced/separated	5 (2.43)
Widowed	4 (1.94)
Religion	
No religious beliefs	181 (87.86)
Buddhism	17 (8.25)
Christianity	6 (2.91)
Others	2 (0.97)
Long-term residence	
Rural	72 (34.95)
City/town	134 (65.05)
Employment status	
Employed	44 (21.36)
Retired	28 (13.59)
Unemployed	134 (65.05)
Caregiver role	
spouse	133 (64.56)
children	32 (15.53)
Others	41 (19.91)
Cancer types	
Oral cavity	18 (8.74)
Pharynx	117 (56.80)
Larynx	20 (9.71)
Nasal cavity	14 (6.80)
Major salivary glands	37 (17.96)
Treatment, ongoing or past 2 months	
Surgery	18 (8.74)
Radiotherapy	32 (15.53)
Surgery and chemoradiation	33 (16.02)
Surgery and radiation	22 (10.68)
Surgery and chemotherapy	9 (4.37)
Chemoradiation	92 (44.66)

### Exploratory factor analysis and ceiling and floor effects

The Kaiser-Mayer-Olkin (KMO) statistic (0.759) exceeded the threshold of 0.5, and Bartlett’s test was significant (chi^2^ = 1051.123, *P* < 0.001), indicating that the data were suitable for factor analysis. The EFA (principal component analysis with direct obnlimin rotation, rotation converged in 9 iterations) was performed. Item 6 had cross loads, and the factor loadings of item 7 and item 9, “Problems with hearing” and “Problems with mobility of neck or shoulders”, were lower on every factor. Item 7 was removed. Then, EFA was repeated with the remaining 10 items. The results were acceptable without cross loading. Bartlett’s sphericity test was statistically significant (chi2 = 987.630, *P* < 0.001), and the KMO value was 0.50. As shown in Table 2, a three-factor structure was identified from the Mandarin version of the SCNS-HNC module, and the cumulative variance contribution rate was 68.266%.

**Table 2:** Floor and ceiling effects and Exploratory Factor Analysis (principal component analysis with Direct Onlimin rotation) of the SCNS-HNC (n = 206)

Item	Lowest Score(%)	Highest Score(%)	Factor loading ^a^			Communalities
			1	2	3	
HNC-specific functioning						
1. Problems with chewing and or swallowing	22.3	17.5	0.698			0.561
2. Problems with dry mouth and/or sticky mucus	11.2	22.8	0.639			0.598
3. Problems with weight (underweight or overweight)	24.3	8.7	0.754			0.580
5. Difficulty speaking	42.2	11.7	0.787			0.625
6. Care of your stoma and/or voice prosthesis	63.1	6.3	0.707			0.611
Lifestyle						
10. Quit smoking	63.6	7.3		0.988		0.945
11. Quit drinking	65.0	6.8		0.971		0.943
Lifestyle						
Nutrition, oral hygiene and mobility of shoulder or neck						
4. To be informed on nutrition	8.3	35.4			0.826	0.699
8. Oral hygiene	12.6	29.1			0.825	0.772
9. Problems with mobility of neck or shoulders	33.5	14.6			0.441	0.491
7. Problems with hearing ^b^	44.7	6.8				
Eigenvalue			3.938	1.757	1.132	6.827
Variance (%)			39.378	17.566	11.323	68.266
KMO = 0.750, Bartlett’s Chi-Square = 987.630^***^						

^a^ Main factor loadings for each item and cross-loading>0.4 are shown

^b^ Item 7 was removed from the EFA

^***^ *P*<0.001

Factor 1 comprised 5 items related to the patient’s head and neck in regard to chewing/swallowing, dry mouth/sticky saliva, speaking, trachea incision or aided articulation device functions and symptoms, and support care for weight problems closely related to transoral eating, accounting for 39.378% of the variance. Factor 2 comprised 2 items regarding the support needs of patients in regard to smoking and alcohol consumption, and the variance contribution rate was 17.566%. Factor 3 comprised 3 items regarding the patients’ needs for nutrition information, oral hygiene, and head and neck movement disorders, with a variance contribution rate of 11.322%. No ceiling effect or floor effect appeared.

### Reliability

As shown in Table 3, the reliability of the scale was investigated from two aspects: internal reliability and retest reliability. According to the analysis, Cronbach’s alpha coefficient ranged from 0.737 to 0.962 in each field of the scale, and the correlation coefficient of the retest assessment was greater than 0.8, except for that of the psychological dimension, which was 0.674.

**Table 3:** Internal consistency and test-retest reliability of the SCNS-SF34 and SCNS-HNC (n = 206 for *α*, *n* = 51 for *r*)

Domains/scales/items	Number of items	Cronbach’s α coefficients	Correlation coefficients r*
SCNS-SF34			
Physical and daily living	5	0.769	0.809
Psychological	10	0.866	0.674
Sexuality	3	0.752	0.885
Health system, information	11	0.891	0.841
Patient care and support	5	0.840	0.835
SCNS-HNC			
HNC-specific functioning	6	0.789	0.870
Nutrition, oral hygiene and mobility of shoulder or neck	3	0.737	0.941
Lifestyle	2	0.962	0.870

* All correlation coefficients r are statistically significant(*P*<0.05)

### Correlation analysis of the structural domain between the SCNS-SF34 and SCNS-HNC

Apart from the correlation coefficient r of health system and information needs and nutrition/oral health/shoulder and neck activity of 0.542, the correlation of the structural domain between the SCNS-HNC and SCNS-SF34 was relatively low (r < 0.5). The structural domain of the SCNS-HNC was positively correlated with that of the SCNS-SF34, as shown in Table 4.

**Table 4:** Correlations between the SCNS-SF34 and SCNS-HNC scales (n = 206)

Item	Physical and daily living	Psychological	Sexuality	Health system, information	Patient care and support
HNC-specific functioning	0.404	0.368	0.375	0.372	0.369
Nutrition, oral hygiene and mobility of shoulder or neck	0.378	0.445	0.125^a^	0.542	0.472
Lifestyle	0.157	0.156	0.302	0.164	0.161

^a^ Not statistically significant (*p* > 0.05); all unmarked are statistically significant

### Clinical validity

As shown in Table 5, the independent-samples *t* test (two-tailed) was used to compare the mean scores of each domain between the two age groups (73 patients 18–60 years old and 33 patients > 60 years old, 124 males and 81 females). Younger patients and males had higher sexual needs than older patients (*P* = 0.007) and female patients (*P* = 0.028).

**Table 5:** Known-groups validity of the SCNS-SF34 and SCNS-HNC (n = 206)

Domain	18 ~ 60 (173)		>60 (33)			Male (124)		Female (81)		
	Mean	SD	Mean	SD	*P* value	Mean	SD	Mean	SD	P value
**SCNS-SF34**										
Physical and daily living	44.57	22.157	40.45	21.986	0.329	42.38	21.073	46.79	23.148	0.160
Psychological	52.76	22.165	51.36	24.789	0.745	51.09	22.370	55.40	22.074	0.176
Sexuality	24.93	25.849	12.03	18.812	0.007^*^	26.11	26.186	18.17	21.896	0.028^*^
Health system information	61.07	22.398	68.87	21.118	0.066^*^	61.60	23.186	63.44	21.200	0.567
Patient care and support	55.49	24.457	60.76	25.468	0.261	55.85	25.277	57.41	23.719	0.658
**SCNS-HNC**										
HNC-specific functioning	41.65	24.179	46.21	25.032	0.324	42.82	23.738	41.67	25.446	0.741
Nutrition, oral hygiene and mobility of shoulder or neck	54.67	28.407	58.33	25.853	0.492	53.29	26.519	58.02	20.117	0.238
Lifestyle	18.86	30.637	17.05	27.744	0.752	27.32	32.680	5.40	19.659	0.000^*^

Note. Higher scores indicate higher levels of supportive care needs

^*^ *P* ≤ 0.05 (statistically significant, independent-samples *t* test, two-tailed)

The older patients had a higher level of need in the health system information domain (*P* = 0.066), and males had higher lifestyle needs than female patients (*P* = 0.000).

## Discussion

In this study, we translated and examined the psychometric properties of the SCNS-HNC in terms of assessing supportive care needs in cancer patients in mainland China, and evaluated the reliability and validity of the SCNS-SF34 in head and neck cancer patients. The Mandarin version of the SCNS-SF34 and SCNS-HNC module can provide an assessment tool for the needs and degrees of needs of head and neck cancer patients in China and provide the basis for appropriate support for patients. On the other hand, it can also promote the development of cross-cultural research comparing the supportive care needs of head and neck cancer patients in different countries.

The original SCNS-HNC module had a two-factor structure, namely, HNC-specific functioning and lifestyle [17]. However, the Mandarin version of the SCNS-HNC module has three dimensions, and the cumulative variance contribution rate was 64.39%. This difference may be related to the different cultural backgrounds and medical environments of China and the Netherlands and the different focus of patients, and it may also be related to the unstable structure of the original scale [17]. In addition, patients with head and neck cancer have specific taste/smell, cough and dyspnoea problems, and the SCNS-HNC module can be updated by further multidisciplinary discussion. The Mandarin version of the SCNS-HNC module had no ceiling effect or floor effect. During the exploratory factor analysis, cross loading occurred. After the discussion between the researchers and the team [32], item 7, “Problems with hearing”, was removed. This may be related to the length of treatment for hearing problems in patients with head and neck cancers. Hearing loss in head and neck cancer patients can occur six months to five years after treatment [33]. Most of the included patients in this study were in the treatment period and may not have hearing problems or the problems may not be serious.

The internal consistency (Cronbach’s α) should be at least 0.7, and test–retest reliability (*r*) should be above 0.8 [34]. The results of the reliability evaluation of the scale in this study showed that the Cronbach’s alpha coefficients of the Mandarin version of the SCNS-HNC module and the Mandarin version of the SCNS-SF34 were > 0.7 (0.737 ~ 0.962) in each domain, suggesting that the internal consistency and stability in different populations of the scale were acceptable. The result was similar to the Cronbach’s alpha coefficient range (0.60 ~ 0.89) of the original SCNS-HNC module, indicating that there was no significant cultural difference in the Mandarin version of the scale, and the Mandarin version of the SCNS-HNC could be understood by patients. Except for the psychological dimension (0.674) of the SCNS-SF34, the test-retest reliability in the other dimensions was > 0.8, suggesting that the time stability of the scale was good. And it was also confirmed in Han’s study in mainland china [23]. The psychological domain consists of 10 items: anxiety, down or depressed feelings, feelings of sadness, fears about the cancer spreading, worry that the results of treatment are beyond your control, uncertainty about the future, feeling in control of your situation, keeping a positive look, feelings about death and dying, and concerns about the worries of those close to you. The low test-retest reliability coefficients may be caused by heterogeneity in the ten items. In addition, this may also be related to the introverted personality of Chinese people [35].

For concurrent validity, except for the correlation coefficient *r* of health system and information needs and nutrition/oral health/shoulder and neck activity of 0.542, the correlation of the structural domain between the SCNS-HNC and SCNS-SF34 was relatively low (r < 0.5). The structural domain of the SCNS-HNC was positively correlated with that of the SCNS-SF34. These results indicated the independence of the SCNS-HNC and SCNS-SF34. In terms of clinical validity, in our study, younger patients had higher sexual needs than older patients (*P* = 0.007), and the older patients had a higher level of need in the health system information domain (*P* = 0.066). Males had higher lifestyle (*P* = 0.000) and sexual (*P* = 0.028) needs than female patients. Except in the domain of sexual needs, the results of our study by age and sex differed from those of previous studies [23, 36, 37]. A possible explanation is that young people are more likely to obtain medical information from online sources than elderly people, so the elderly patients have a higher demand for medical information [38]. In regard to lifestyle, most females lead relatively healthy lifestyles [39], while males may need more help with their lifestyles, especially in regard to smoking and alcohol consumption.

## Conclusions

In summary, the Mandarin version of the SCNS-HNC scale was Sinicized in this study, and the reliability and validity of the Mandarin version of the SCNS-SF34 and SCNS-HNC module were tested. The results showed that the Mandarin version of the SCNS-SF34 and SCNS-HNC module had good reliability and validity in head and neck cancer patients. The Mandarin version of the SCNS-HNC module is concise, clear, easy to understand and accepted by patients, and it can be used with SCNS-SF34 to investigate the supportive care needs of Chinese patients with head and neck cancer and provide a basis for medical staff to understand patients’ needs and adopt targeted medical care support in a timely manner. Although this study followed the principle of a sample size 10- to 25-times the number of items, a limitation of this study conducted in patients with a tumour treated in a hospital in Chengdu in Sichuan Province was the use of a convenience sampling method, which may affect the representativeness of the survey population. Due to the limited time and number of researchers, the retest interval of this study is only two to three days. Therefore, it is suggested that follow-up studies expand the sample and scope, adopt more reasonable sampling methods, extend the interval time between repeated measurements appropriately, and further verify and develop the Mandarin version of the SCNS-HNC module.

## Data Availability

The datasets generated and/or analysed in the current study are not publicly available due to individual privacy but are available from the corresponding author on reasonable request.

## Abbreviations

SCNS-SF34: Supportive Care Needs Survey Short-Form 34
SCNS-HNC: Head and Neck Cancer-specific Supportive Care Needs
